# Zinc finger protein ZPR9 functions as an activator of AMPK-related serine/threonine kinase MPK38/MELK involved in ASK1/TGF-β/p53 signaling pathways

**DOI:** 10.1038/srep42502

**Published:** 2017-02-14

**Authors:** Hyun-A Seong, Ravi Manoharan, Hyunjung Ha

**Affiliations:** 1 Department of Biochemistry, School of Biological Sciences, Chungbuk National University, Cheongju 28644, Republic of Korea; 2 National Center for Nanoscience and Nanotechnology, University of Madras, Guindy Campus, Chennai 600025, India

## Abstract

Murine protein serine-threonine kinase 38 (MPK38), an AMP‐activated protein kinase (AMPK)-related kinase, has been implicated in the induction of apoptosis signal-regulating kinase 1 (ASK1)-, transforming growth factor-β (TGF‐β)-, and p53-mediated activity involved in metabolic homeostasis. Here, zinc finger protein ZPR9 was found to be an activator of MPK38. The association of MPK38 and ZPR9 was mediated by cysteine residues present in each of these two proteins, Cys^269^ and Cys^286^ of MPK38 and Cys^305^ and Cys^308^ of ZPR9. MPK38 phosphorylated ZPR9 at Thr^252^. Wild‐type ZPR9, but not the ZPR9 mutant T252A, enhanced ASK1, TGF‐β, and p53 function by stabilizing MPK38. The requirement of ZPR9 Thr^252^ phosphorylation was validated using CRISPR/Cas9-mediated ZPR9 (T252A) knockin cell lines. The knockdown of endogenous ZPR9 showed an opposite trend, resulting in the inhibition of MPK38‐dependent ASK1, TGF‐β, and p53 function. This effect was also demonstrated in mouse embryonic fibroblast (MEF) cells that were haploinsufficient (+/−) for ZPR9, NIH 3T3 cells with inducible knockdown of ZPR9, and CRISPR/Cas9-mediated ZPR9 knockout cells. Furthermore, high-fat diet (HFD)-fed mice displayed reduced MPK38 kinase activity and ZPR9 expression compared to that in mice on control chow, suggesting that ZPR9 acts as a physiological activator of MPK38 that may participate in obesity.

Emerging evidence has implicated ASK1/TGF‐β/Smad3 signaling in the pathogenesis of obesity-associated metabolic diseases. For instance, ASK1 signaling has been shown to associate with TGF‐β signaling and to contribute to the improvement of glucose and lipid metabolism in genetically and diet-induced obese mice^1^. TGF‐β signaling has been shown to be involved in numerous metabolic processes, including systemic glucose and lipid metabolism, pancreatic β-cell function, adipocyte differentiation, adipocytokine secretion and inflammation^2^. In addition, evidence of interplay between p53 and mechanistic target of rapamycin (mTOR) signaling, glucose and lipid metabolism, or mitochondrial maintenance suggested that p53 also plays a crucial role in the regulation of cellular metabolic homeostasis^3^.

MPK38, otherwise known as maternal embryonic leucine zipper kinase (MELK), was an AMPK‐related serine-threonine kinase that was highly conserved across different species. It regulated a variety of cellular processes, including the cell cycle, cell proliferation, spliceosome assembly, carcinogenesis, stem cell self‐renewal, apoptosis, and numerous signal transduction pathways^4^,^5^,^6^,^7^,^8^,^9^,^10^,^11^,^12^,^13^. MPK38 was activated by different stimuli, including H_2_O_2_, tumor necrosis factor-α (TNF-α), thapsigargin, ionomycin, TGF-β1, 5-fluorouracil (5FU), and doxorubicin (Dox), that trigger ASK1, TGF-β, and p53 signaling pathways^5^,^14^,^15^,^16^. The intracellular signaling proteins ASK1, Smads, and p53 were recently found to interact with MPK38 *in vivo*, indicating a critical role for MPK38 in redox-sensitive ASK1, TGF‐β, and p53 signaling pathways^17^,^18^,^19^. Activated MPK38 phosphorylated ASK1 and increased ASK1 activity^5^. Upon activation, MPK38 also stimulated redox-sensitive TGF-β- and p53-mediated signaling through direct interaction with and phosphorylation of Smads and p53, respectively^15^,^16^. One possible regulatory mechanism for MPK38 activity involves thioredoxin (Trx), which was recently reported to negatively regulate MPK38-induced ASK1, TGF‐β, and p53 function in a phosphorylation-dependent manner^20^. However, the regulation of MPK38 activity is poorly understood.

The zinc finger protein ZPR9 was initially identified as a physiological substrate of MPK38^21^. It was mainly localized in the cytoplasm, but its interacting partner MPK38 redirected the subcellular localization of ZPR9 from the cytoplasm to the nucleus through direct protein interaction^21^. ZPR9 was also able to stimulate B-myb transcriptional activity via physical interaction with B-myb^22^. ZPR9 was recently shown to enhance H_2_O_2_-induced apoptosis via a redox-dependent interaction with ASK1, suggesting that ZPR9 is a positive regulator of redox-sensitive ASK1 signaling^14^. However, the physiological role of ZPR9 is still unclear.

In the present study, we demonstrate that ZPR9 stimulates MPK38 activity and function via a redox-dependent interaction, resulting in the stabilization of MPK38. We also show that ZPR9 phosphorylation at Thr^252^ by MPK38 plays a central role in the positive regulation of MPK38-mediated activation of ASK1, TGF‐β, and p53 signaling by ZPR9.

## Results

### ZPR9 promotes MPK38 kinase activity via a redox-dependent interaction and phosphorylation

**Figure 1:**
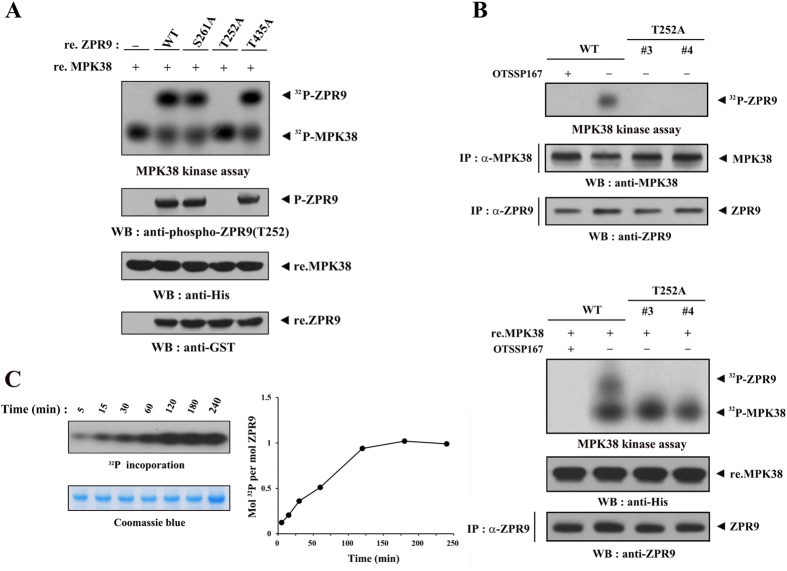
ZPR9 phosphorylation at Thr^252^ by MPK38. (**A**) To identify the MPK38 phosphorylation site on ZPR9, recombinant MPK38 proteins (re.MPK38) were screened in an *in vitro* kinase assay using recombinant wild-type ZPR9 or one of the substitution ZPR9 mutants (S261A, T252A, and T435A) as the substrate. (**B**) *In vitro* kinase assays were performed using MPK38 immunoprecipitates, which were obtained from cell lysates of wild-type and clonal CRISPR/Cas9 ZPR9 (T252A) KI isolates (clones #3 and #4), treated with (+) or without (−) OTSSP167 (1 μM, 2 h), a MPK38-specific inhibitor, in the presence of ZPR9 immunoprecipitates as the substrate. Recombinant MPK38 proteins (lower panels), which were expressed in bacterial cells^15^, treated with (+) or without (−) OTSSP167 were also used instead of the MPK38 immunoprecipitates (upper panels) in the MPK38 kinase assay. (**C**) Stoichiometry of ZPR9 phosphorylation by MPK38. ZPR9 was phosphorylated by MPK38 for the indicated time periods. The *in vitro* kinase reactions were terminated by the addition of SDS sample buffer, the products were separated by SDS-PAGE and exposed to X-ray film for ~30 min (left upper panel). Coomassie staining was employed to see if the ZPR9 load is equal (left lower panel). Gel slices containing labelled ZPR9 were excised and the amount of radioactivity was measured by the liquid scintillation counting, and the moles of ^32^P incorporated per mole ZPR9 were calculated and plotted as a function of time (right panel). Stoichiometric analysis was actually conducted as described^33^. Minus (−) or plus (+) indicates absence or presence, respectively. WT, wild-type; ^32^P, ^32^P incorporation; P, phosphorylated; IP, immunoprecipitation; re., recombinant; WB, western blot.

**Figure 2:**
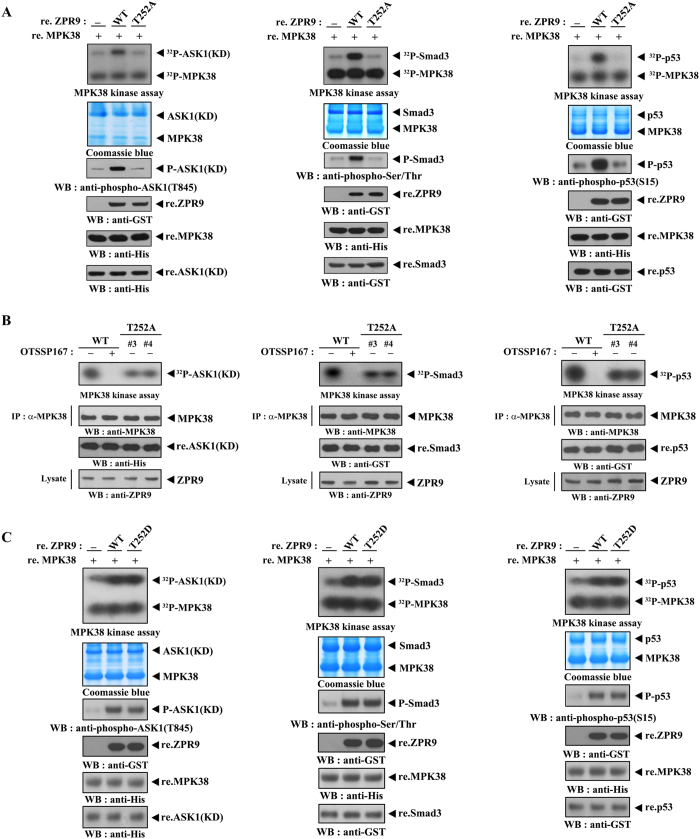
Stimulation of MPK38 kinase activity by ZPR9 in a phosphorylation-dependent manner. Recombinant MPK38 proteins were screened in *in vitro* kinase assays using recombinant kinase-dead (KD) ASK1^5^, Smad3^15^, or p53^16^ protein as the substrate in the presence or absence of recombinant WT, T252A, or T252D ZPR9 (**A** and **C**), or *in vitro* kinase assays using endogenous MPK38 proteins from wild-type and ZPR9 T252A KI cells were performed in the presence or absence of OTSSP167 (**B**). Stoichiometry of MPK38 phosphorylation of ASK1, Smad3, and p53 was shown in Supplementary Fig. S6.

We recently demonstrated that ASK1 is stimulated by MPK38 through direct interaction with and phosphorylation by MPK38^2^. Additionally, ASK1 activity was positively regulated by a redox‐dependent interaction with ZPR9^14^, a substrate of MPK38^21^. Based on these findings, we reasoned that ZPR9 may directly or indirectly regulate MPK38 activity. An alignment analysis with the MPK38/AMPK consensus sequence Hyd‐(X,Basic)XX(S/T)XXX‐Hyd (Hyd = hydrophobic residue, Basic = basic residue)^23^ suggested three putative MPK38 phosphorylation sites (Thr^252^, Ser^261^, and Thr^435^) on ZPR9. The ZPR9 T252A mutant, but not the S261A or T435A mutant, completely abolished MPK38‐mediated phosphorylation of ZPR9 (Fig. 1A), indicating that Thr^252^ is the MPK38 phosphorylation site on ZPR9. MPK38-mediated phosphorylation of ZPR9 at Thr^252^ *in vivo* was validated in ZPR9 (T252A) knockin 3T3-L1 cell lines generated by the CRISPR/Cas9 system. MPK38 kinase reactions including OTSSP167, a MPK38-specific inhibitor^24^, showed clearly that both endogenous and recombinant MPK38 proteins directly phosphorylate ZPR9 at Thr^252^ (Fig. 1B). In addition, a stoichiometric analysis of ZPR9 phosphorylation by MPK38 supported that MPK38 phosphorylates only one residue (Thr^252^) in ZPR9 (Fig. 1C). We then measured the kinase activity of MPK38 in the presence or absence of ZPR9 using *in vitro* kinase assays with recombinant MPK38 proteins (Fig. 2A). The coexpression of ZPR9 markedly increased the kinase activity of MPK38, indicating that ZPR9 positively regulates MPK38 activity. To investigate whether the ability of ZPR9 to stimulate MPK38 kinase activity requires both direct interaction with and phosphorylation by MPK38, we also screened the ZPR9 T252A mutant in *in vitro* kinase assays. The coexpression of the T252A mutant had no effect on MPK38 kinase activity compared to that of the control, which contained wild‐type MPK38 recombinant protein alone. Consistently, the kinase activity of MPK38 was considerably decreased in ZPR9 knockout and (T252A) knockin 3T3-L1 cells compared to control wild-type 3T3-L1 cells (Fig. 2B and Supplementary Fig. S1). The treatment of wild-type 3T3-L1 cells with OTSSP167 also displayed no MK38 kinase activity. These results support again a central role for ZPR9 phosphorylation at Thr^252^ by MPK38 in the ZPR9-mediated stimulation of MPK38 kinase activity. On the contrary, the phosphomimetic mutation T252D displayed high levels of MPK38 kinase activity similar to that of the wild-type ZPR9 (Fig. 2C), providing additional validation of the requirement of ZPR9 phosphorylation at Thr^252^ in the stimulation of MPK38 kinase activity. Together, these results suggest a critical role for both a redox-dependent interaction with and phosphorylation by MPK38 in the ZPR9-mediated stimulation of MPK38 kinase activity.

### ASK1/TGF-β/p53 signals increase the redox-dependent ZPR9–MPK38 interaction

**Figure 3:**
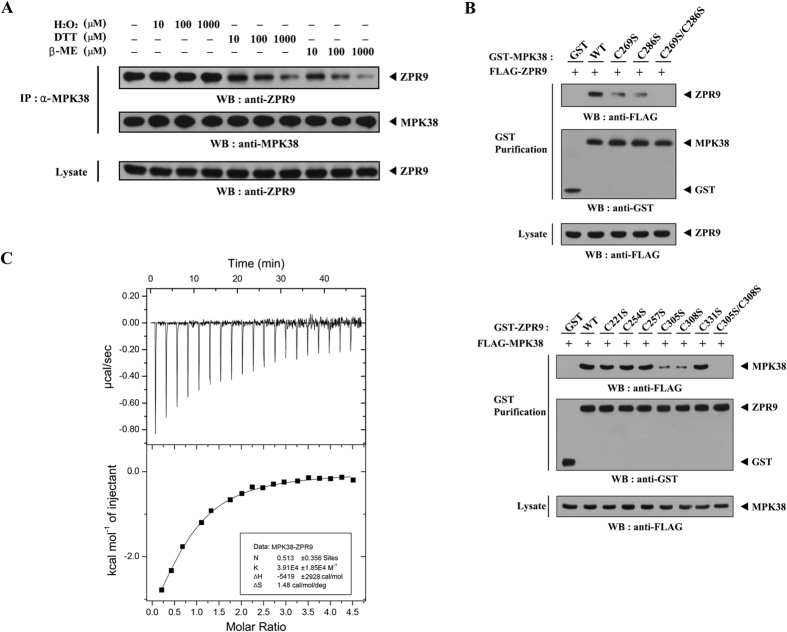
Redox‐dependency of the interaction between MPK38 and ZPR9. (**A**) HEK293 cell lysates were treated with the indicated concentrations of H_2_O_2_, DTT, and β‐ME on ice for 0.5–1 h and then subjected to immunoprecipitation using an anti‐MPK38 antibody (IP:α-MPK38). Immune complexes were then analyzed for the presence of ZPR9 by immunoblot analysis using an anti‐ZPR9 antibody. (**B**) To examine the effects of a single amino acid substitution of the cysteine residue on MPK38-ZPR9 complex formation, HEK293 cells transfected with the indicated expression vectors were lysed and GST precipitates (GST purification) were then analyzed for MPK38-ZPR9 complex formation by immunoblot analysis using an anti‐FLAG antibody. (**C**) ITC analysis of MPK38-ZPR9 interaction. Data were analyzed using MicroCal Origin^TM^ to determine stoichiometry (N) and binding affinity (1/K) of the interaction between MPK38 and ZPR9. Recombinant His-tagged wild-type MPK38 and ZPR9 proteins were used for ITC experiments.

Given that ZPR9 interacts with ASK1 in a redox-dependent manner^14^, we analyzed the possibility of a redox-dependent interaction between ZPR9 and MPK38. To this end, an *in vivo* binding assay was performed using lysates from HEK293 cells treated with the oxidant or reductants to measure the level of endogenous ZPR9–MPK38 complex formation. Dithiothreitol (DTT) or β‐mercaptoethanol (β‐ME) treatment markedly decreased ZPR9-MPK38 complex formation compared to that in the untreated control, whereas H_2_O_2_ had no effect (Fig. 3A). We next performed an *in vivo* binding assay to identify the specific cysteine residues of MPK38 for ZPR9 binding using seven cysteine‐to‐serine amino acid substitution MPK38 mutants (C169S, C204S, C269S, C286S, C339S, C377S, and C269S/C286S). As a result, ZPR9 interacted weakly with two MPK38 mutants, C269S and C286S, and no binding was detected in the presence of the C269S/C286S double mutant (Fig. 3B, upper panels, and Supplementary Fig. S2), indicating that both Cys^269^ and Cys^286^ of MPK38 are responsible for ZPR9 binding. To identify the specific cysteine residues of ZPR9 for MPK38 binding, similar experiments were performed using seven cysteine‐to‐serine amino acid substitution ZPR9 mutants (C221S, C254S, C257S, C305S, C308S, C331S, and C305S/C308S). MPK38 interacted weakly with the two ZPR9 mutants, C305S and C308S, and no complex formation was detected in the presence of the C305S/C308S double mutant (Fig. 3B, lower panels), indicating that both Cys^305^ and Cys^308^ of ZPR9 are required for MPK38 binding. In addition, an isothermal titration calorimetry (ITC) experiment using recombinant wild-type MPK38 and ZPR9 proteins exhibited a 1: 2 stoichiometry in the binding of ZPR9 to each MPK38 monomer (Fig. 3C). These results clearly suggest that a redox-dependent interaction between ZPR9 and MPK38 occurs in cells.

**Figure 4:**
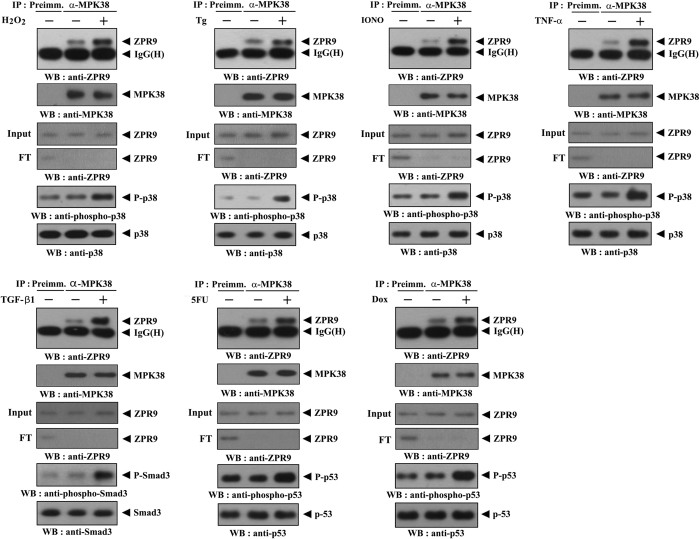
Increased MPK38-ZPR9 complex formation by ASK1, TGF‐β, and p53 signals. HEK293 cells were treated with (+) or without (−) stimuli as follows: H_2_O_2_ (2 mM, 30 min), thapsigargin (Tg: 20 μM, 30 min), ionomycin (IONO: 1 μM, 24 h), TNF‐α (500 ng/ml, 30 min), TGF‐β1 (100 ng/ml, 20 h), 5-fluorouracil (5FU: 0.38 mM, 30 h), or doxorubicin (Dox: 100 ng/ml, 24 h). The cell lysates were then subjected to immunoprecipitation with either rabbit preimmune serum (IP:Preimm.) or an anti‐MPK38 antibody (IP:α-MPK38), followed by immunoblot analysis using an anti‐ZPR9 antibody (top panels). An equal level of immunoprecipitated MPK38 was verified by immunoblotting with an anti-MPK38 antibody (2nd panels). As a control, the expression levels of ZPR9 in the total cell lysate (Input) and flow-through (FT), a supernatant fraction of IP samples, were analyzed by immunoblotting with an anti-ZPR9 antibody (3rd and 4th panels). Input and FT indicate the samples on 5% of volume used for IP and the samples on 5% of flow-through fraction of IP samples, respectively. Activation of ASK1/TGF-β/p53 signaling in the presence (+) or absence (−) of apoptotic stimuli was measured by immunoblot analysis with anti‐phospho-specific antibodies for p38 Thr^180^/Tyr^182^, Smad3 Ser^423^/Ser^425^, and p53 Ser^15^, respectively (upper and lower panels, second from the bottom).

Previous studies revealed that the binding of MPK38 to its binding partners, such as ASK1 and Smads, is modulated in response to various oxidative stress agents and signals, including H_2_O_2_, TNF‐α, endoplasmic reticulum stress induced by thapsigargin, calcium overload induced by ionomycin, TGF‐β1, 5FU, and Dox, that activate ASK1, TGF-β, and p53 signaling pathways^5^,^15^. To examine whether these signals also influence the interaction of ZPR9 with MPK38, we investigated the endogenous complex formation between ZPR9 and MPK38 in HEK293 cells exposed to these signals. All ASK1/TGF-β/p53 signals enhanced complex formation between endogenous ZPR9 and MPK38 compared to that in untreated control cells (Fig. 4). These results suggest a positive role of ZPR9 in the regulation of redox‐sensitive ASK1, TGF‐β, and p53 signaling pathways induced by MPK38.

### ZPR9 enhances c-Jun N-terminal kinase (JNK)‐mediated transcription and H_2_O_2_-mediated apoptosis induced by MPK38

**Figure 5:**
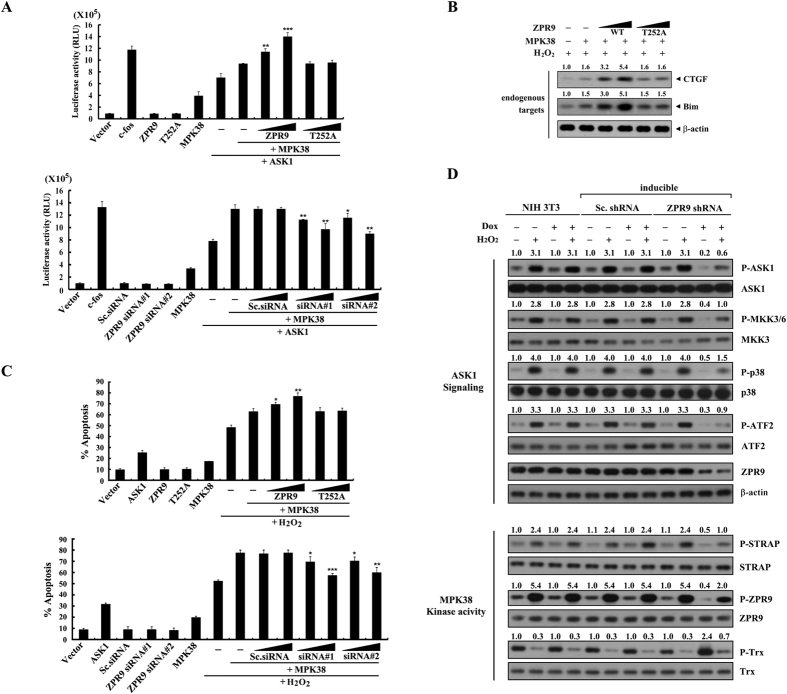
Stimulation of MPK38‐mediated ASK1 downstream signaling by ZPR9. (**A**) 293 T cells were transfected with increasing concentrations of expression vectors for WT and T252A ZPR9 (0.5 and 1 μg), ZPR9-specific siRNAs (#1 and #2, each at 100 and 200 nM), control scrambled siRNAs (100 and 200 nM), WT MPK38 (1 μg), WT ASK1 (0.6 μg), c‐fos (0.6 μg), or the AP‐1-luciferase plasmid (0.2 μg) as indicated. *p < 0.05, **p < 0.01, ***p < 0.001 compared with MPK38 alone in the presence of ASK1. (**B**) Transcriptional responses were complemented under the same conditions by immunoblot analysis using anti-CTGF and anti-Bim antibodies for endogenous ASK1/AP-1 targets in the presence of H_2_O_2_ (2 mM, 30 min). (**C**) HEK293 cells were transfected with increasing concentrations of expression vectors for WT and T252A ZPR9 (0.5 and 1 μg), ZPR9-specific siRNAs (#1 and #2, each at 100 and 200 nM), control scrambled siRNAs (100 and 200 nM), WT MPK38 (upper panel, 0.5 μg; lower panel, 1 μg), or WT ASK1 (1 μg) as indicated in the presence or absence of H_2_O_2_ (1 mM, 9 h). Cells exposed only to H_2_O_2_ were used as a positive control. *p < 0.05, **p < 0.01, ***p < 0.001 compared with MPK38 alone in the presence of H_2_O_2_. (**D**) NIH 3T3 cells harboring stably integrated pSingle‐tTS‐shRNA containing a ZPR9‐specific shRNA (inducible ZPR9 shRNA) or a nonspecific scrambled shRNA (inducible Sc. shRNA), as well as parental NIH 3T3 cells (NIH 3T3), treated with (+) or without (−) H_2_O_2_ (2 mM, 30 min) in the presence or absence of doxycycline (Dox: 1 μg/ml, 72 h) were lysed and used for immunoprecipitation. The immunoprecipitates were analyzed by immunoblot analysis using anti‐phospho-specific antibodies for ASK1 Thr^845^ (Thr^838^ in human), MKK3/6 Ser^189/207^, p38 Thr^180^/Tyr^182^, and ATF2 Thr^71^. Inducible silencing of endogenous ZPR9 by doxycycline treatment was confirmed by immunoblot analysis using an anti‐ZPR9 antibody (upper panels, second from the bottom). The effects of ZPR9 on MPK38 kinase activity were also determined by immunoblot analysis^20^,^34^ using anti‐phospho-specific antibodies for STRAP Ser^188^, ZPR9 Thr^252^, and Trx Thr^76^. Sc., scrambled.

Given that MPK38 stimulates ASK1-mediated activator protein 1 (AP‐1) transcriptional activity^5^, we assessed whether ZPR9 affects ASK1-mediated transactivation induced by MPK38 using an AP-1-luciferase reporter. Wild‐type ZPR9 increased MPK38‐induced AP‐1 transcriptional activity in a dose-dependent manner, whereas the ZPR9 T252A mutant had no effect (Fig. 5A, upper panel). Consistently, knockdown of endogenous ZPR9 using two different ZPR9-specific small interfering RNAs (siRNAs) significantly decreased MPK38‐induced AP‐1 transcriptional activity in a dose-dependent manner (Fig. 5A, lower panel). These transcriptional responses were also verified by immunoblot analysis of the endogenous ASK1/AP-1 targets, including connective tissue growth factor (CTGF) and Bcl-2 interacting mediator (Bim) (Fig. 5B). These results indicate that ZPR9 positively regulates MPK38‐induced AP‐1 transactivation in a MPK38 phosphorylation-dependent manner.

We further conducted a green fluorescent protein (GFP)-based apoptosis assay to examine the effects of ZPR9 on H_2_O_2_-mediated cell death induced by MPK38 in HEK293 cells. ZPR9 resulted in a significant increase in H_2_O_2_‐mediated cell death in a dose-dependent manner, whereas the ZPR9 T252A mutant had no effect (Fig. 5C, upper panel). This result was also confirmed in a ZPR9-knockdown system induced by ZPR9-specific siRNAs (Fig. 5C, lower panel). We then assessed the role of ZPR9 in MPK38‐dependent ASK1 signaling using NIH 3T3 cells harboring an inducible ZPR9 short hairpin RNA (shRNA). Knockdown of endogenous ZPR9 markedly decreased the phosphorylation levels of ASK1 downstream targets and the kinase activity of MPK38 compared to parental NIH 3T3 cells and control cells expressing a scrambled shRNA (Fig. 5D and Supplementary Fig. S3). Together, these results suggest a positive role of ZPR9 in MPK38‐dependent ASK1 signaling, probably through an upregulation of MPK38 kinase activity.

### ZPR9 enhances TGF‐β-mediated transcription and apoptosis induced by MPK38

**Figure 6:**
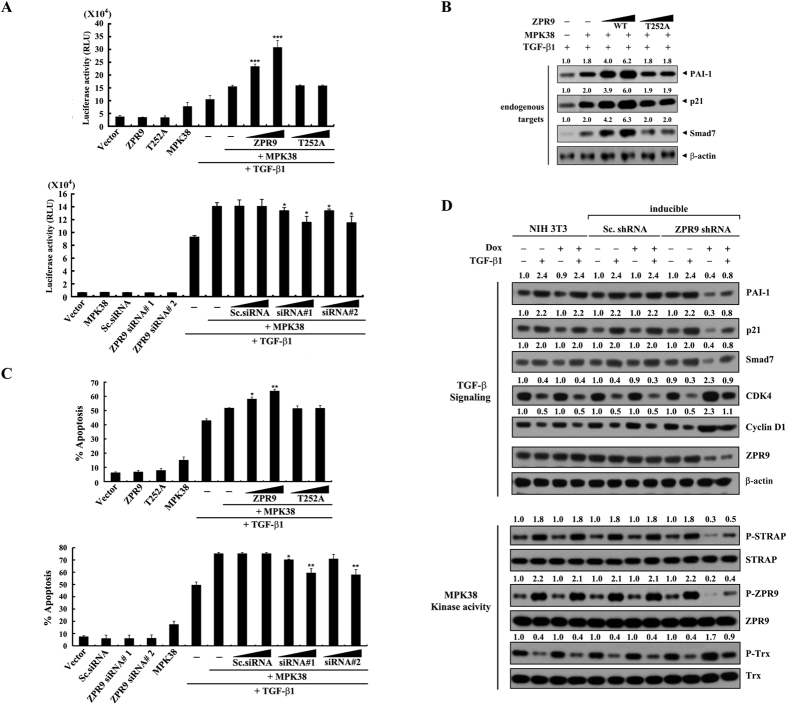
Stimulation of MPK38‐mediated TGF-β signaling by ZPR9. (**A**) To examine MPK38-induced TGF‐β-mediated transcription, Hep3B cells were transfected with increasing concentrations of expression vectors for WT and T252A ZPR9 (0.5 and 1 μg), ZPR9-specific siRNAs (#1 and #2, each at 100 and 200 nM), control scrambled siRNAs (100 and 200 nM), WT MPK38 (1 μg), or the p3TP‐Lux plasmid (0.2 μg) as indicated in the presence or absence of TGF‐β1 (100 pM). *p < 0.05, ***p < 0.001 compared with MPK38 alone in the presence of TGF-β1. (**B**) Transcriptional responses were complemented under the same conditions by immunoblot analysis using the indicated antibodies for endogenous TGF‐β targets (PAI-1, p21, and Smad7). (**C**) To determine the effects of ZPR9 on MPK38-induced TGF‐β-mediated apoptosis, HaCaT cells were transfected with increasing concentrations of expression vectors for WT and T252A ZPR9 (0.5 and 1 μg), ZPR9-specific siRNAs (#1 and #2, each at 100 and 200 nM), control scrambled siRNAs (100 and 200 nM), or WT MPK38 (upper panel, 0.5 μg; lower panel, 1 μg) as indicated with an expression vector encoding GFP (1 μg). After treatment of the transfected cells with TGF‐β1 (2 ng/ml, 20 h), apoptotic cell death was determined using a GFP system^5^,^26^. *p < 0.05, **p < 0.01 compared with MPK38 alone in the presence of TGF-β1. (**D**) To determine the effects of ZPR9 on TGF‐β signaling, inducible ZPR9 shRNA, inducible Sc. shRNA, or parental NIH 3T3 cells were treated with TGF‐β1 (2 ng/ml, 20 h), and the cell lysates were then analyzed by immunoblot analysis using the indicated antibodies. β‐Actin was used as a loading control. The effects of ZPR9 on MPK38 kinase activity were determined under the same conditions by immunoblot analysis using the anti‐phospho-specific antibodies shown in Fig. 5D. The relative expression levels of the TGF‐β targets and the relative phosphorylation levels of the MPK38 substrates were quantified by densitometry, and the fold-increase relative to untreated control parental NIH 3T3 cells was presented for each protein. The results represent the means ± S.E. of three independent experiments.

Because MPK38 activated TGF‐β signaling through direct interaction with and phosphorylation of Smads^15^, we also investigated the effects of ZPR9 on MPK38‐induced TGF‐β transcriptional activity using a p3TP-luciferase reporter in Hep3B cells transfected with MPK38 and increasing concentrations of expression plasmids for wild‐type and mutant (T252A) ZPR9. Wild‐type ZPR9 significantly increased MPK38‐induced TGF‐β transcriptional activity in a dose-dependent manner, whereas the ZPR9 T252A mutant had no effect (Fig. 6A, upper panel). Consistently, MPK38‐induced TGF‐β transcriptional activity decreased dose-dependently in ZPR9-knockdown Hep3B cells compared to that in Hep3B cells expressing MPK38 alone or control cells expressing a scrambled siRNA (Fig. 6A, lower panel). The transcriptional responses were also confirmed in similar experiments by immunoblot analysis of endogenous TGF‐β targets, including plasminogen activator inhibitor‐1 (PAI‐1), cyclin‐dependent kinase inhibitor p21^Cip1^, and Smad7 (Fig. 6B). These data indicate that ZPR9 also plays a major role in stimulating MPK38-induced TGF‐β transcriptional activity.

We next examined whether ZPR9 can influence TGF‐β-dependent cell death induced by MPK38. The coexpression of wild‐type ZPR9 resulted in a dose‐dependent increase in apoptosis in HaCaT cells compared to that in control cells expressing MPK38 alone in the presence of TGF‐β1, whereas the T252A mutant had no effect (Fig. 6C, upper panel). Consistent with this, knockdown of endogenous ZPR9 by ZPR9‐specific siRNAs dose‐dependently decreased TGF‐β-dependent apoptosis induced by MPK38 (Fig. 6C, lower panel). A similar finding was also noted in the inducible ZPR9 shRNA cell line, as determined by immunoblot analysis of TGF‐β targets^25^, including PAI‐1, p21^Cip1^, Smad7, cyclin-dependent kinase 4 (CDK4), and cyclin D1 (Fig. 6D, upper panels, and Supplementary Fig. S3). Concurrently, a downregulation of MPK38 kinase activity was also observed after the knockdown of endogenous ZPR9 (Fig. 6D, lower panels). These results indicate that ZPR9 stimulates MPK38‐induced TGF‐β activity and function in a MPK38 phosphorylation-dependent manner by upregulating MPK38 kinase activity.

### ZPR9 enhances p53-mediated transcription and apoptosis induced by MPK38

**Figure 7:**
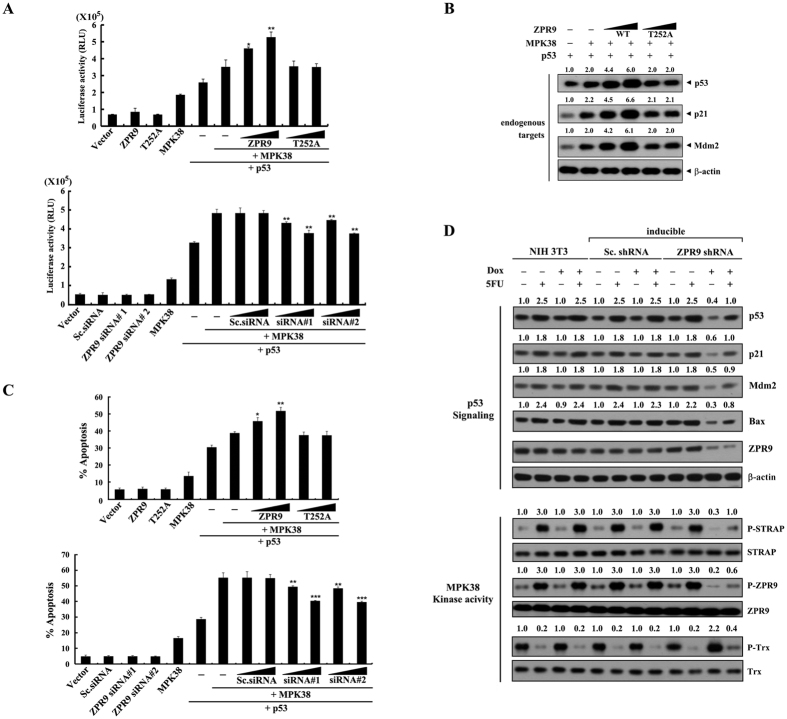
Stimulation of MPK38‐mediated p53 signaling by ZPR9. (**A**) To examine MPK38-induced p53-mediated transcription, MCF7 cells were transfected with increasing concentrations of expression vectors for WT and T252A ZPR9 (0.5 and 1 μg) or ZPR9-specific siRNAs (#1 and #2, each at 100 and 200 nM), control scrambled siRNAs (100 and 200 nM), WT MPK38 (1 μg), and the p53-luciferase plasmid (0.2 μg) as indicated in the presence or absence of p53 (0.3 μg). *p < 0.05, **p < 0.01 compared with MPK38 alone in the presence of p53. (**B**) Transcriptional responses were complemented under the same conditions by immunoblot analysis using the indicated antibodies for endogenous p53 targets (p53, p21, and Mdm2). (**C**) To determine the effects of ZPR9 on MPK38-induced p53-mediated apoptosis, MCF7 cells were transfected with increasing concentrations of expression vectors for WT and T252A ZPR9 (0.5 and 1 μg), ZPR9-specific siRNAs (#1 and #2, each at 100 and 200 nM), control scrambled siRNAs (100 and 200 nM), or WT MPK38 (upper panel, 0.5 μg; lower panel, 1 μg) as indicated together with an expression vector encoding GFP (1 μg) in the presence or absence of p53 (1 μg). Apoptotic cell death was then determined using a GFP system. *p < 0.05, **p < 0.01, ***p < 0.001 compared with MPK38 alone in the presence of p53. (**D**) To determine the effects of ZPR9 on p53 signaling, inducible ZPR9 shRNA, inducible Sc. shRNA, or parental NIH 3T3 cells were treated with (+) or without (−) 5FU (0.38 mM, 30 h) and then analyzed by immunoblot analysis using the indicated antibodies. The relative expression levels of the p53 targets were quantified by densitometry, and the fold-increase relative to untreated control parental NIH 3T3 cells was presented for each protein. The effects of ZPR9 on MPK38 kinase activity were determined under the same conditions by immunoblot analysis using the anti‐phospho-specific antibodies shown in Fig. 5D. The results represent the means ± S.E. of three independent experiments.

Because MPK38 interacted with p53 and stimulated p53 signaling^16^, we determined whether ZPR9 has an effect on MPK38‐induced p53 activity. To do this, p53-induced transcription was measured by a luciferase reporter assay in the presence of wild-type and mutant (T252A) ZPR9. Expression of wild‐type ZPR9 significantly increased MPK38‐dependent p53 transcriptional activity in a dose-dependent manner, whereas the T252A mutant had no effect (Fig. 7A, upper panel). This result was also confirmed by knockdown experiments using ZPR9‐specific siRNAs (Fig. 7A, lower panel). The transcriptional responses were also verified by immunoblot analysis of endogenous p53 targets, including p53, p21^Cip1^, and mouse double minute 2 homolog (Mdm2), under the same conditions (Fig. 7B). These results indicate that ZPR9 stimulates MPK38‐induced p53 transactivation in a MPK38 phosphorylation-dependent manner. We next examined whether ZPR9 has a similar effect on MPK38-dependent p53-mediated apoptosis using the GFP-based apoptosis assay. As expected, coexpression of wild‐type ZPR9 resulted in a dose‐dependent increase in apoptosis compared to control cells expressing MPK38 alone in the presence of p53, whereas the T252A mutant had no effect (Fig. 7C, upper panel). Consistently, knockdown of endogenous ZPR9 by ZPR9‐specific siRNAs dose‐dependently decreased the p53-mediated apoptosis induced by MPK38 (Fig. 7C, lower panel). Similar results were also obtained in the inducible ZPR9 shRNA cell line, in which knockdown of endogenous ZPR9 markedly decreased the expression level of p53 targets, including p53, p21^Cip1^, Mdm2, and Bax, and the kinase activity of MPK38 compared to either parental NIH 3T3 cells or control cells expressing a scrambled shRNA (Fig. 7D and Supplementary Fig. S3). Together, these data indicate that ZPR9 interacts with MPK38 and also contributes to the stimulation of MPK38-dependent p53 activity and function through upregulation of MPK38 kinase activity.

### ZPR9 increases the stability of MPK38 and the complex formation between MPK38 and ASK1, Smad3, and p53

**Figure 8:**
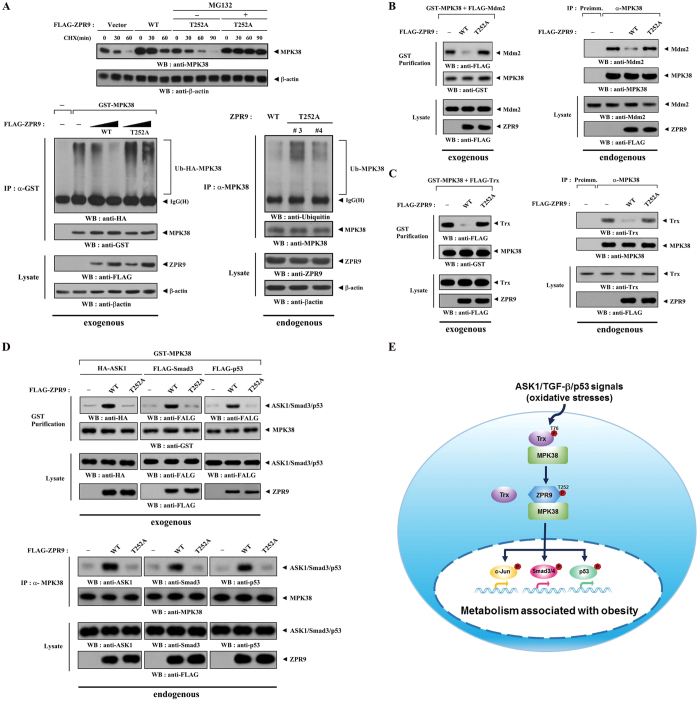
Increased MPK38 stability and complex formation between MPK38 and ASK1, Smad3, and p53 by ZPR9. (**A**) MPK38 protein stability was assessed by immunoblot analysis using an anti‐MPK38 antibody (upper panels). Time intervals indicate the number of minutes after treatment with cycloheximide (CHX) (20 μg/ml) alone or with MG132 (10 μM). To determine MPK38 ubiquitination, p53‐null HCT116 cells were transfected with increasing concentrations of expression vectors for WT and T252A ZPR9 (0.5 and 1 μg), HA‐tagged ubiquitin (Ub), and GST-tagged MPK38 as indicated (left lower panels). The ubiquitination of endogenous MPK38 was determined using wild-type and ZPR9 T252A KI cells (right lower panels). (**B**) To determine MPK38-Mdm2 complex formation, HEK293 cells were transfected with the indicated expression vectors. The cell lysates were subjected to precipitation with glutathione‐Sepharose beads (GST purification), followed by immunoblot analysis using an anti‐FLAG antibody (left panels). HEK293 cells were transfected with the indicated expression vectors. MPK38 immunoprecipitates (IP:α-MPK38) were subjected to immunoblot analysis with an anti-Mdm2 antibody to assess the association between endogenous MPK38 and Mdm2 (right panels). (**C**) HEK293 cells were transfected with the indicated combinations of expression vectors. The cell lysates were then subjected to precipitation with glutathione-Sepharose beads, and the association between exogenous MPK38 and Trx was assessed by immunoblot analysis using an anti-FLAG antibody (left panels). To determine the association between endogenous MPK38 and Trx, MPK38 immunoprecipitates were subjected to immunoblot analysis with an anti-Trx antibody (right panels). (**D**) HEK293 cells were transfected with the indicated combinations of expression vectors. The exogenous complex formation was assessed by immunoblot analysis using anti-HA and anti-FLAG antibodies (upper panels). To measure the endogenous complex formation, MPK38 immunoprecipitates were subjected to immunoblot analysis with the indicated antibodies (lower panels). (**E**) A model for the role of ZPR9 in the regulation of MPK38-dependent ASK1, TGF-β, and p53 signaling. ASK1/TGF-β/p53 signals dissociate Trx, an inactivator of MPK38, from the MPK38-Trx complex and induce MPK38-ZPR9 complex formation, leading to the stimulation of ASK1/TGF-β/p53 signaling pathways. This eventually contributes to the amelioration of obesity-associated metabolic conditions in HFD-fed mice.

To address how ZPR9 stimulates MPK38-dependent ASK1, TGF‐β, and p53 signaling, we first performed immunoblot analysis using lysates from HEK293 cells transfected with expression vectors for wild-type and mutant (T252A) ZPR9 to examine the effects of ZPR9 on the stability of MPK38. Wild‐type ZPR9 increased the stability of MPK38 compared to that in control cells expressing the empty vector, whereas the T252A mutant had no effect (Fig. 8A, upper panels), indicating that ZPR9 phosphorylation at Thr^252^ plays a critical role in MPK38 stabilization. On the contrary, treatment of the HEK293 cells expressing the T252A mutant with both cycloheximide and MG132, a proteasomal inhibitor, led to an increased stability of MPK38 compared to that in control cells expressing the T252A mutant in the absence of MG132 (Fig. 8A, upper panels). These results confirm the role of the proteasome pathway in the degradation of MPK38 in cells. Consistent with this finding, ZPR9 decreased both exogenously and endogenously the ubiquitination of MPK38 in a MPK38 phosphorylation-dependent manner (Fig. 8A, lower panels). We also determined whether ZPR9 can regulate MPK38 degradation through Mdm2 *in vitro* and *in vivo* using *in vivo* binding assays because MPK38 interacts with Mdm2^20^. Compared to control cells coexpressing MPK38 and Mdm2 in the absence of ZPR9, coexpression of wild‐type ZPR9 markedly decreased MPK38-Mdm2 complex formation, whereas the T252A mutant had no effect (Fig. 8B, left panels). Consistently, a significant decrease in complex formation between endogenous MPK38 and Mdm2 was also observed in the presence of wild-type ZPR9, but not the T252A mutant (Fig. 8B, right panels). These results indicate that ZPR9 stabilizes MPK38 in a MPK38 phosphorylation-dependent manner by inhibiting Mdm2‐mediated MPK38 ubiquitination. To further understand the mechanism whereby ZPR9 promotes the stability of MPK38, we also investigated the effects of ZPR9 on MPK38-Trx complex formation because Trx functioned as a destabilizer of MPK38^20^. Coexpression of wild‐type ZPR9 significantly decreased both exogenous and endogenous MPK38-Trx complex formation in a MPK38 phosphorylation-dependent manner, whereas the T252A mutant had no effect (Fig. 8C).

Given that Trx decreases MPK38 complex formation with ASK1, Smad3, and p53 to inhibit MPK38-dependent ASK1, TGF-β, and p53 signaling^20^, we finally examined whether ZPR9, similar to Trx, can influence the physical interaction between MPK38 and ASK1, Smad3, and p53. Compared to control HEK293 cells in the absence of ZPR9, the expression of wild-type ZPR9 resulted in a significant increase in both exogenous and endogenous complex formation between MPK38 and ASK1, Smad3, and p53, whereas the T252A mutant had no effect on the binding of these key signaling proteins (Fig. 8D), suggesting an important role for complex formation between MPK38 and ASK1, Smad3, and p53 in the ZPR9-mediated stimulation of MPK38-dependent ASK1, TGF-β, and p53 signaling. Taken together, these data indicate that ZPR9 positively regulates MPK38-dependent ASK1, TGF-β, and p53 function by stabilizing MPK38, leading to the increased complex formation between MPK38 and ASK1, Smad3, and p53.

### ZPR9 improves obesity-associated metabolic disturbances in mice by upregulating MPK38 kinase activity

**Figure 9:**
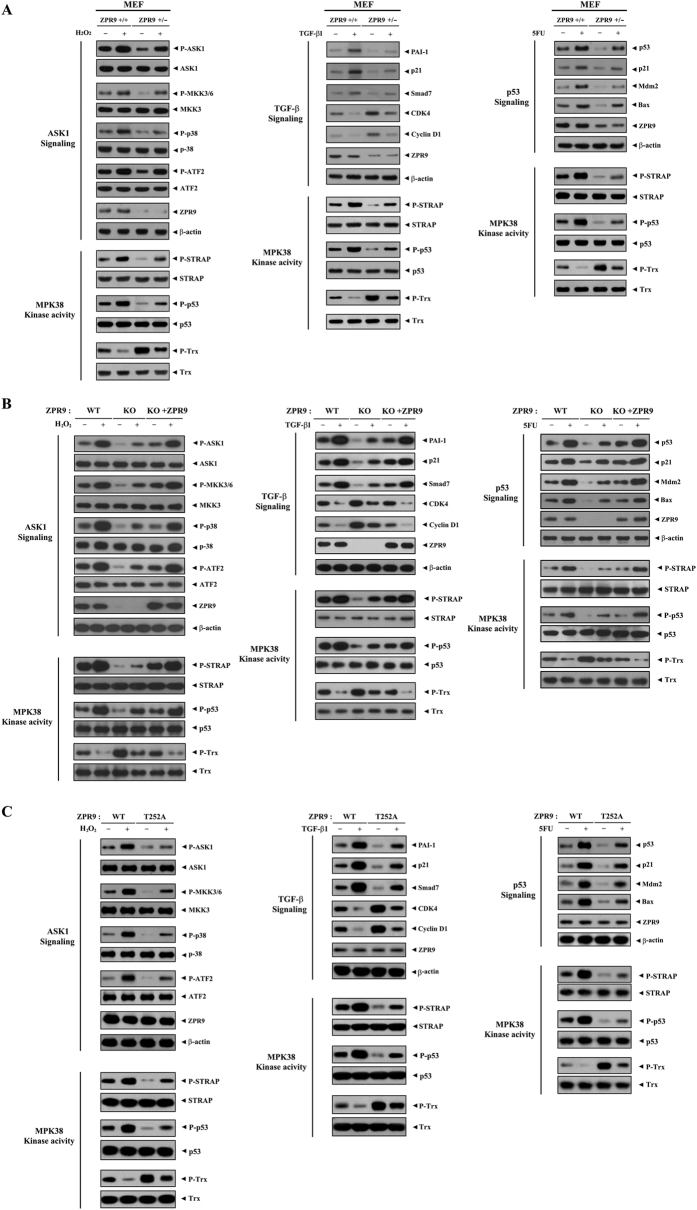
Downregulation of ASK1/TGF-β/p53 signaling activation and MPK38 kinase activity by ZPR9 knockdown, knockout, and knockin. MEF cells that were intact (+/+) or haploinsufficient (+/−) for ZPR9 (**A**), as well as ZPR9 KO HEK293 cells generated using CRISPR/Cas9 genome-editing (KO) and ZPR9 KO HEK293 cells transfected with ~3 μg of ZPR9 (KO + ZPR9) (**B**), were incubated with (+) or without (−) H_2_O_2_ (5 mM, 30 min), TGF‐β1 (100 ng/ml, 20 h), or 5FU (0.38 mM, 30 h), followed by immunoblot analysis with the indicated antibodies shown in Figs 4, 5, 6, 7. Activation of ASK1/TGF-β/p53 signaling in WT ZPR9 and CRISPR/Cas9 ZPR9 (T252A) KI 3T3-L1 cells cultured in the presence (+) or absence (−) of apoptotic stimuli as indicated in A was measured by immunoblot analysis with the indicated antibodies (**C**). The MPK38 kinase activity was also measured under the same conditions by immunoblot analysis using anti‐phospho-specific antibodies shown in Figs 4 and 5D.

To verify the physiological role of ZPR9 in the stimulation of MPK38-dependent ASK1, TGF-β, and p53 signaling pathways in mice, we first performed immunoblot analysis using MEF cells that were intact (+/+) or haploinsufficient (+/−) for ZPR9. As expected, the redox-sensitive activation of ASK1/TGF-β/p53 signaling in MEF^ZPR9+/−^ cells was much lower than that in MEF^ZPR9+/+^ cells (Fig. 9A, upper panels), indicating a critical role for ZPR9 in the activation of ASK1/TGF-β/p53 downstream signaling *in vivo*. MPK38 kinase activity in MEF^ZPR9+/−^ cells was also much lower than that in MEF^ZPR9+/+^ cells (Fig. 9A, lower panels). Similar results were also obtained for the activation levels of redox-sensitive ASK1/TGF-β/p53 signaling in CRISPR/Cas9 ZPR9 KO HEK293 cells (Fig. 9B). Consistently, the rescue of ZPR9 expression by transfection with the KO gene ZPR9 in ZPR9 KO cells (KO + ZPR9) markedly increased the activation levels of ASK1/TGF-β/p53 signaling, as well as the kinase activity of MPK38, compared to those in control ZPR9 KO cells (Fig. 9B). Indeed, the ZPR9 KO cells were validated by no phosphorylation of ZPR9 by MPK38 due to the absence of endogenous ZPR9 (Supplementary Fig. S4). We also confirmed the requirement of MPK38-mediated ZPR9 phosphorylation at Thr^252^ in the ZPR9-mediated stimulation of ASK1, TGF-β, and p53 signaling pathways using CRISPR/Cas9 ZPR9 knockin (T252A) 3T3-L1 cells (Fig. 9C). These results indicate that ZPR9 functions as a physiological activator of MPK38, thereby stimulating redox-sensitive ASK1-, TGF-β-, and p53-mediated function in a MPK38 phosphorylation-dependent manner.

**Figure 10:**
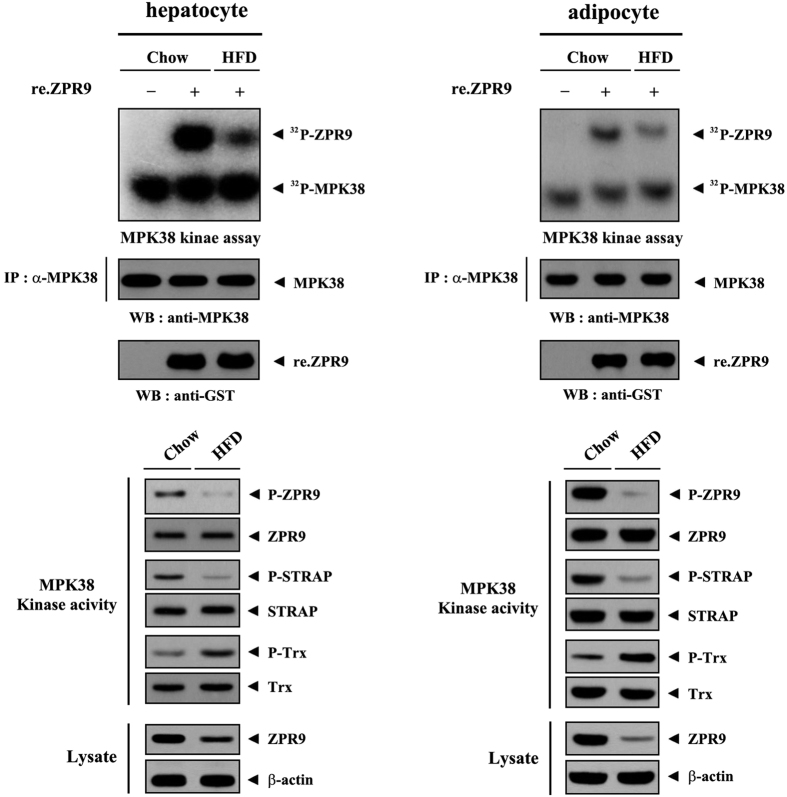
Downregulation of MPK38 kinase activity and ZPR9 expression in HFD-fed mice. Hepatocytes or adipocytes obtained from C57BL/6 J mice on a standard chow diet or HFD were subjected to immunoprecipitation with an anti-MPK38 antibody, followed by *in vitro* kinase assays using MPK38 immunoprecipitates (IP:α-MPK38) in the presence or absence of recombinant ZPR9 as the substrate (upper panels), or the cell lysates were subjected to immunoprecipitation with the indicated antibodies followed by immunoblot analysis using anti‐phospho-specific antibodies shown in Fig. 5D to determine the endogenous level of MPK38 kinase activity (lower panels). The endogenous ZPR9 expression level was also determined by immunoblot analysis with an anti-ZPR9 antibody (lower panels, second from the bottom). β-actin was used as a loading control. These experiments were performed at least three independent times with similar results.

Because of the reduction in the activation of redox-dependent ASK1/TGF-β signaling and ZPR9 expression in both genetic and diet-induced obese mice compared to control wild-type and chow mice^1^, we next assessed whether ZPR9 can improve obesity-associated metabolic disturbances in diet-induced obese mice by stimulating MPK38 activity (Fig. 10). Indeed, MPK38 kinase activity was downregulated in HFD-fed mice, as determined by results of *in vitro* kinase assays and immunoblot analysis, in both hepatocytes and adipocytes compared to that in control mice on a standard diet, leading to reduced activation of redox-sensitive ASK1/TGF-β/p53 signaling. These results reveal that ZPR9 contributes to the coordinated activation of MPK38-dependent ASK1/TGF-β/p53 signaling, which might ameliorate obesity and its associated metabolic conditions in mice.

## Discussion

We previously showed that MPK38 interacts with ASK1, Smads, or p53 and activates the respective signaling pathways. We also showed that ZPR9 interacts with ASK1 and stimulates its activity^5^,^14^,^15^,^16^, suggesting a potential role for ZPR9 in regulating MPK38 activity and governing redox-sensitive ASK1/TGF-β/p53 signaling. In this study, we identified a redox-dependent interaction between ZPR9 and MPK38 that stimulates MPK38‐dependent ASK1, TGF‐β, and p53 activity (Fig. 3). Additionally, MPK38-mediated ZPR9 phosphorylation at Thr^252^ played a critical role in the activation of MPK38 (Fig. 2). The C-terminal domain of MPK38 that interacted with ZPR9 was also required for Trx, a negative regulator of MPK38^20^. Thus, the binding of ZPR9 to the C-terminal domain of MPK38 may contribute to the removal of Trx from the MPK38-Trx complex, leading to the stimulation of MPK38-dependent activity. A redox-dependent interaction with and phosphorylation by MPK38 were responsible for ZPR9-mediated stimulation of MPK38 activity and function, as demonstrated by results of the *in vitro* kinase assays (Fig. 2), luciferase reporter assays (Figs 5, 6 and 7), and apoptosis assays (Figs 5, 6 and 7). In contrast to wild-type ZPR9, the T252A mutant, which lacks the MPK38 phosphorylation site (see Fig. 1A), had no effect on MPK38‐dependent ASK1, TGF‐β, and p53 signaling pathways (Figs 5, 6 and 7). We also demonstrated that MPK38‐mediated phosphorylation of ZPR9 at Thr^252^ plays a critical role in modulating the physical association of MPK38 with ASK1^5^, Smad3^15^, and p53^16^, which function as key signaling molecules for redox-sensitive ASK1, TGF‐β, and p53 signaling pathways. As shown in Fig. 8D, wild-type ZPR9 markedly increased complex formation between MPK38 and ASK1, Smad3, and p53, whereas the T252A mutant had no effect on the binding of MPK38 to its signaling targets, supporting again a key role for ZPR9 phosphorylation at Thr^252^ in the regulation of MPK38-dependent signaling pathways involving ASK1, TGF‐β, and p53.

To understand the mechanism whereby ZPR9 stimulates MPK38 activity, the effects of ZPR9 on MPK38 stability and ubiquitination were examined. Wild‐type ZPR9 increased MPK38 protein stability, whereas the T252A mutant had no effect (Fig. 8A). These results suggest that ZPR9 increases both the stability of MPK38 and the complex formation between MPK38 and its signaling targets (ASK1, Smad3, and p53) through a redox-dependent interaction with and phosphorylation by MPK38, leading to the stimulation of redox‐sensitive ASK1, TGF‐β, and p53 signaling pathways. As it was recently reported that ZPR9 functions as a positive component of a multi-protein complex linking redox-sensitive ASK1 and TGF‐β signaling pathways, which improve glucose and lipid metabolism in obese mice^1^, we also reasoned that the downregulation of MPK38 activity in obese mice likely occurs through the reduced expression of ZPR9. As expected, low ZPR9 expression and reduced MPK38 kinase activity were observed in both hepatocytes and adipocytes isolated from HFD-fed mice compared to those isolated from mice fed a standard chow diet (Fig. 10). These data suggest a model in which ZPR9 promotes the association of MPK38 with key signaling targets, such as ASK1, Smad3, and p53, that play pivotal roles in inducing redox-sensitive ASK1, TGF‐β, and p53 signaling pathways in stressed cells, probably through the removal of Trx, a negative regulator of MPK38, from the MPK38-Trx complex. Subsequently, the elevated activation levels of ASK1, TGF‐β, and p53 signaling contribute to the amelioration of glucose and lipid metabolism in obese mice (Fig. 8E).

However, our present results do not rule out the possibility that direct interactions between ZPR9 and key signaling molecules (ASK1, Smad3, and p53) in redox-sensitive ASK1, TGF‐β, and p53 signaling pathways also play a role in regulating their respective functions. In fact, our recent results showed that ZPR9 stimulates ASK1- and TGF‐β-mediated function via direct interactions with ASK1 and Smad3, respectively^1^,^14^. In addition, we also observed that the ability of ZPR9 to stimulate p53-mediated signaling is dependent on the redox-dependent interaction between ZPR9 and p53 (Supplementary Fig. S5A). These findings, together with data obtained in Fig. 8D, indicate that ZPR9 both directly and indirectly contributes to the stimulation of redox-sensitive ASK1, TGF‐β, and p53 signaling pathways. Furthermore, the inhibitory effect of MPK38 on PDK1 activity^26^ that plays an important role in cell survival signaling through direct phosphorylation of PDK1 at Thr^354^ prompted us to examine whether ZPR9, acting as a physiological substrate of MPK38, is also involved in the regulation of cell proliferation, in addition to cell death signaling pathways that involve ASK1^14^, TGF‐β^1^, and p53 (Supplementary Fig. S5B). In fact, our previous study^14^ showed that ZPR9 decreases the activity of PDK1 by regulating Akt phosphorylation at Thr^308^ and Ser^473^. These results could contribute to a better understanding of the mechanism by which ZPR9 potentiates apoptotic cell death that is connected to many physiological functions, including obesity.

In conclusion, our results indicate that zinc finger protein ZPR9 positively regulates redox‐sensitive ASK1, TGF‐β, and p53 signaling pathways involved in obesity-associated metabolic functions by the stabilization of MPK38 and that this effect of ZPR9 is strictly dependent on MPK38-mediated phosphorylation. A novel function of ZPR9 as an activator of redox-sensitive ASK1, TGF‐β, and p53 signaling pathways also provides a more complete picture of how cellular signaling pathways are regulated by intracellular redox state.

## Methods

### Cell culture, inducible ZPR9 shRNA cell line, MEF cells, antibodies, plasmids, chemicals, ZPR9 siRNAs, and animal experiments

HEK293, NIH 3T3, MCF7, Hep3B, 293 T, p53‐null HCT116, and HaCaT cells were grown in Dulbecco’s modified Eagle’s medium supplemented with 10% fetal bovine serum (Gibco BRL) at 37 °C in a humidified incubator containing 5% CO_2_^16^. Inducible ZPR9 shRNA NIH 3T3 stable clones were screened in the presence of G418 (450 μg/ml, 14 days) until all control NIH 3T3 cells died as described previously^15^. MEF^ZPR9+/−^ has been described previously^1^. The use of anti-FLAG (M2), anti-His, anti-hemagglutinin (HA), anti-ASK1, anti-MPK38, anti-glutathione-S-transferase (GST), anti-MKK3, anti-CDK4, anti-cyclin D1, anti-PAI-1, anti-p21, anti-activating transcription factor 2 (ATF2), anti-phospho-MKK3/6(S189/207), anti-p38, anti-phospho-p38(T180/Y182), anti-phospho-Smad3(S423/S425), anti-phospho-ATF2(T71), anti-phospho-ASK1(T845), anti-p53(DO-1), anti-phospho-p53(S15), anti-Mdm2, anti-Bax, and anti-β-actin antibodies has been described previously^5^,^16^,^25^,^27^. Anti-Trx, anti-CTGF, and anti-Bim antibodies were purchased from Santa Cruz Biotechnology, Inc. (Santa Cruz, CA, USA). The anti-phospho-ZPR9(T252) antibody was raised in a rabbit against the synthetic phosphopeptide antigen GAIPITLCLFC, where T represents phosphothreonine (Young In Frontier, Seoul, Korea). Mouse or rabbit IgG TrueBlot (Rockland, Limerick, PA, USA) was used as secondary antibody for some experiments. Plasmids encoding MPK38, ASK1, Smad3, Mdm2, p53, p3TP-luciferase reporter, AP-1-luciferase reporter, p53-luciferase reporter, and c-fos have been described previously^5^,^16^,^27^,^28^. MG132 was purchased from BIOMOL International, L.P. (Plymouth Meeting, PA, USA), and OTSSP167 was from ChemScene (Monmouth Junction, NJ)^24^,^29^. The use of ZPR9 siRNAs (#1, 5′-GCGACGUACACCUGCAUAA-3′; #2, 5′-GCACCGUUUGCAGUAAGAA-3′) has been described previously^14^. Animal experiments were performed in accordance with the approved animal protocols and the guidelines established by the Ethics Review Committee of Chungbuk National University for Animal Experiments (CBNUA-966-16-02).

### MPK38-mediated phosphorylation-defective and phosphomimetic ZPR9 mutants

Four serine (or threonine)-to-alanine (or aspartate) amino acid substitution ZPR9 mutants were generated by polymerase chain reaction (PCR) as described previously^5^,^14^. Wild-type ZPR9 was used as the template for amplification with either the ZPR9 forward primer (5′-GC GAATTCATGGCGACGTACACCTGCATA-3′), containing an EcoR1 site (underlined), or the reverse primer (5′-GCCTCGAGTCAGAATCTCACTTGGACCCG-3′), containing an XhoI site (underlined), in conjunction with one of the following mutant primer pairs: 5′-TTTTGTTCCCATCATGCCAGCTCGCTGATGAAG-3′ (S261A, forward) and 5′-CTTCATCAGCGAGCTGGCATGATGGGAACAAAA-3′ (S261A, reverse), 5′-GGTGCCATCCCTATCGCAGACTGCTTATTTTGT-3′ (T252A, forward) and 5′-ACAAAATAAGCAGTCTGCGATAGGGATGGCACC-3′ (T252A, reverse), 5′-GGATGGACTGGCAGCGCAGGAGCGGCTCTTATG-3′ (T435A, forward) and 5′-CATAAGAGCCGCTCTGCGCTGCCAGTCCATCC-3′ (T435A, reverse), and 5′-GGTGCCATCCCTATCGATGACTGCTTATTTTGT-3′ (T252D, forward) and 5′-ACAAAATAAGCAGTCATCGATAGGGATGGCACC-3′ (T252D, reverse). To generate pGEX4T-1-ZPR9 (S261A), (T252A), (T435A), and (T252D), the amplified PCR products were digested with EcoRI and XhoI and then ligated into the corresponding sites of the pGEX4T-1 vector.

### Cysteine-to-serine substitution ZPR9 and MPK38 mutants

Two cysteine-to-serine amino acid substitution MPK38 mutants were generated by PCR using wild-type MPK38 (for the C269S mutant) or the C269S single site mutant of MPK38 (for the C269S/C286S double mutant) as the template for amplification with either the MPK38 forward (5′-CGCGGATCCATGAAAGATTATGAC-3′, EcoRI site is underlined) or reverse (5′-CGCCTCGAGCATCTTGCAGCCAGA-3′, XhoI site is underlined) primer, in conjunction with one of the following mutant primer pairs: 5′-CAAGATTACAGCAGCC CCGTGGAGTGG-3′ (C269S, forward) and 5′-CCACTCCACGGGGCTGCTGTAATCTTG-3′ (C269S, reverse) or 5′-CACCTCGATGATGATAGCGTAACAGAACTTTCT-3′ (C269S/C286S, forward) and 5′-AGAAAGTTCTGTTACGCTATCATCATCGAGGTG-3′ (C269S/C286S, reverse). To generate GST-tagged MPK38 mutants, pGEX4T-1 (C269S) and (C269S/C286S) were digested with BamHI and NotI and then ligated into the corresponding sites of pEBG. The generation of GST-tagged ZPR9 mutants (C221S, C254S, C257S, C305S, C308S, C331S, and C305S/C308S) and the other MPK38 mutant (C286S) has been described previously^14^,^20^.

### *In vivo* binding assays and *in vitro* kinase assays for MPK38

*In vivo* binding assays were performed using HEK293 cells transiently transfected with the indicated expression vectors as described previously^5^,^14^. To measure MPK38 kinase activity, GST-tagged MPK38, purified by glutathione-Sepharose beads from cells expressing GST-MPK38, or recombinant His-tagged MPK38 was washed three times with lysis buffer and then twice with kinase buffer (50 mM HEPES, pH 7.4, 1 mM DTT, and 10 mM MgCl_2_) before screening in an *in vitro* kinase assay (37 °C, 15 min) using recombinant ZPR9, Smad3, kinase-dead (KD) ASK1, and p53 as the substrates^5^,^20^. The reaction mixtures were separated by sodium dodecyl sulfate polyacrylamide gel electrophoresis (SDS-PAGE) and analyzed by autoradiography.

### Generation of ZPR9 (T252A) knockin (KI) and knockout (KO) cell lines

Genomic mutations in 3T3-L1 or HEK293 cells were generated using the CRISPR/Cas9 system as described previously^30^,^31^. In brief, single-guide (sg) RNAs were designed to target the genomic areas adjacent to the ZPR9 mutation site (target sequence, 5′-GCTGAGGAAGGCCCACCCCT-3′). Two complementary oligos (5′-CACCGGCTGAGGAAGGCCCACCCCT-3′ and 5′-AAACAGGGGTGGGCCTTCCTCAGCC-3′) containing the ZPR9 guide sequence and Bbs1 ligation adapters were synthesized by Bioneer Ltd. (Cheongwon, Korea). The annealed oligos were ligated into the Bbs1-digested pX458 vector (Addgene plasmid no. 48138) using the Quick-Ligation system (New England BioLabs, Ipswich, MA, USA). To generate the ZPR9 (T252A) KI cell line, 3T3-L1 cells were cultured in a 24-well plate at ~60% confluence and cotransfected with 1 μg of ZPR9 sg RNA plasmid and pUC19 ZPR9(T252A) using Lipofectamine 2000 (Invitrogen, Carlsbad, CA, USA) or WelFect-Ex™ Plus (WelGENE, Daegu, Korea). To generate the ZPR9 KO cell line, HEK293 cells were transfected with 1 μg of ZPR9 sg RNA plasmid without pUC19. Forty-eight hours after transfection, the cells were trypsinized and diluted in medium for single-cell seeding in a 96-well plate, and GFP-positive cells were screened, followed by genomic DNA extraction. Exon 2 of ZPR9 was amplified by PCR using a ZPR9-specific PCR primer pair as follows: 5′-ATTGGGAAGATATTGATTCTGATGAAGA-3′ (forward) and 5′-CCAAGTATTTAATCAGT CCCTTAATATCTG-3′ (reverse). The PCR products were A-tailed and cloned into the pGEM-T Easy vector (Promega, Madison, WI, USA). The individual ZPR9 (T252A) KI clones were then confirmed by DNA sequencing (Bioneer Ltd., Cheongwon, Korea). On the other hand, the ZPR9 KO clones were validated by immunoblotting with an anti-ZPR9 antibody.

### Isothermal titration calorimetry

The ITC measurements were carried out using an Auto-iTC_200_ Microcalorimeter (Korea Basic Science Institute, Ochang, Korea) at 25 °C in a buffer containing 50 mM HEPES (pH 7.4), 10 mM MgCl_2_, and 0.01% sodium lauroyl sarcosinate. Wild-type ZPR9 (40 μM) was titrated into a calorimeter cell containing 3 μM wild-type MPK38 with 2 μL injections. The ITC data were analyzed using the MicroCal Origin^TM^ software.

### Ubiquitination and luciferase reporter assays

Ubiquitination assays were performed using p53-null HCT116 or wild-type and ZPR9 T252A KI cells transfected with or without expression plasmids encoding MPK38, wild-type and mutant ZPR9 (T252A), and HA-tagged ubiquitin as described previously^26^. For luciferase reporter assays, 293 T, Hep3B, or MCF7 cells were transfected using WelFect-Ex™ Plus with the AP-1-, p3TP-, or p53-luciferase reporter plasmid, along with the indicated expression vectors in the presence or absence of c-fos, TGF-β1, or p53. Luciferase activity was assessed using the dual luciferase assay kit (Promega, Madison, WI, USA) as described previously^5^. This experiment was performed at least three independent times.

### Apoptosis assay

Apoptosis was detected using the GFP system in HEK293, HaCaT, or MCF7 cells transfected with the indicated expression vectors as described previously^5^. In brief, apoptosis was assessed by fluorescent microscopy of 4′,6′-diamidino-2-phenylindole dihydrochloride (DAPI)-stained nuclei of GFP-positive cells. The percentage of apoptotic cells was calculated as the number of GFP-positive cells with apoptotic nuclei divided by the total number of GFP-positive cells.

### Isolation of primary hepatocytes and adipocytes

Primary hepatocytes were prepared from the livers of HFD- and standard chow diet-fed mice using the collagenase perfusion method as previously described^32^ with minor modifications. The mice were anesthetized, and their abdomens were surgically opened. Each liver was perfused at a flow rate of 25 ml/min for 10 min via the hepatic portal vein with pre-warmed (37 °C) Krebs-Henseleit buffer without Ca^2+^ and SO_4_^2−^ (115 mM NaCl, 25 mM NaHCO_3_, 5.9 mM KCl, 1.18 mM MgCl_2_, 1.23 mM NaH_2_PO_4_, and 6 mM glucose), followed by perfusion at a flow rate of 20 ml/min for 10 min with pre-warmed (37 °C) collagenase type II solution (20 mg of collagenase type II in 100 ml of Krebs-Henseleit buffer containing 0.1 mM CaCl_2_ and 3% BSA, but not SO_4_^2−^). The liver was transferred into a Petri dish containing 5 ml of collagenase type II solution, cut into small pieces, and finally flushed with ice-cold Krebs-Henseleit buffer containing 1.2 mM Na_2_SO_4_ and 1.25 mM CaCl_2_ using a household sieve. The cell suspension was then filtered through a 70 μm pore size nylon filter strainer into a 50 ml tube. Approximately 10 ml of ice-cold DMEM was added to the filtered cell suspension, which was subsequently centrifuged at 500 rpm for 2 min at 4 °C. The supernatant was aspirated, and the remaining hepatocyte pellet was washed three times with 10 ml of ice-cold DMEM. The cell suspension was finally adjusted to a density of 17% iodixanol in Krebs-Henseleit buffer containing 1.25 mM CaCl_2_ and 1.2 mM Na_2_SO_4_. Hepatocytes were isolated after centrifugation at 1,400 rpm for 20 min at 4 °C.

To isolate primary adipocytes from the epididymal fat depots of HFD- and standard chow diet-fed mice, adipose tissues in HEPES buffer (pH 7.4, containing 5 mM D-glucose, 2% BSA, 135 mM NaCl, 2.2 mM CaCl_2_, 1.25 mM MgSO_4_, 0.45 mM KH_2_PO_4_, 2.17 mM Na_2_HPO_4_, and 10 mM HEPES) were digested with collagenase type I (1.25 mg of collagenase type I in 1 ml of HEPES buffer) at 37 °C for 30 min by gentle shaking. The resulting cell suspension was then filtered through a 400 μm pore size nylon mesh to remove undigested tissues. The filtered cell suspension was incubated with DMEM supplemented with 1% FBS at 37 °C for 40 min before plating.

### Statistical analysis

Data are presented as the means ± standard error and are representative of at least three independent experiments. The statistical analysis was conducted using GraphPad Prism software. Multiple comparisons were performed by one-way ANOVA, followed by Tukey’s post hoc test.

## Additional Information

**How to cite this article**: Seong, H.-A. *et al*. Zinc finger protein ZPR9 functions as an activator of AMPK-related serine/threonine kinase MPK38/MELK involved in ASK1/TGF-β/p53 signaling pathways. *Sci. Rep.* **7**, 42502; doi: 10.1038/srep42502 (2017).

**Publisher's note:** Springer Nature remains neutral with regard to jurisdictional claims in published maps and institutional affiliations.

## Supplementary Material

Supplementary Information
