# Supplementary material for: Zinc finger protein ZPR9 functions as an activator of AMPK-related serine/threonine kinase MPK38/MELK involved in ASK1/TGF-β/p53 signaling pathways
Source: Sci Rep. 2017 Feb 14;7:42502. doi: 10.1038/srep42502 (PMC5307367; doi:10.1038/srep42502)
Supplement: Supplementary Information [file srep42502-s1.pdf]

Zinc finger protein ZPR9 functions as an activator of AMPK-related serine/threonine kinase MPK38/MELK involved in ASK1/TGF-β/p53 signaling pathways

Hyun-A Seong[1], Ravi Manoharan[2], and Hyunjung Ha[1,*]

[1]Department of Biochemistry, School of Biological Sciences, Chungbuk National University, Cheongju 28644, Republic of Korea

[2]National Center for Nanoscience and Nanotechnology, University of Madras, Guindy Campus, Chennai 600025, India

*To whom correspondence should be addressed: Hyunjung Ha (hyunha@chungbuk.ac.kr) Department of Biochemistry, School of Biological Sciences, Chungbuk National University, Cheongju 28644, Republic of Korea

(Phone) 82-43-261-3233

(E-mail) hyunha@chungbuk.ac.kr

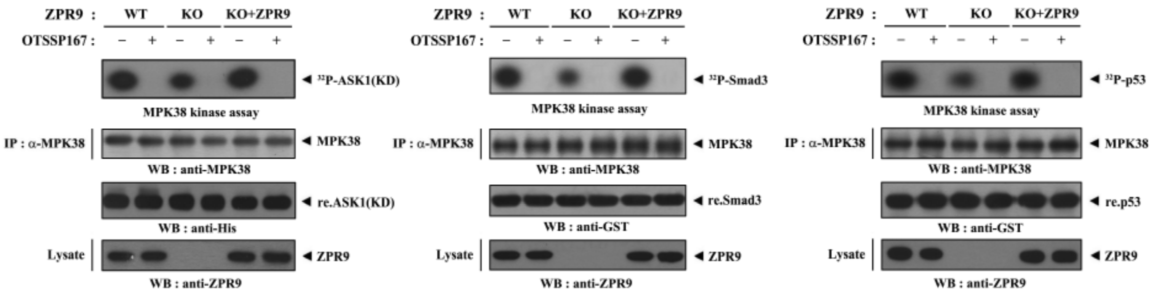

Supplementary Figure S1. Rescue of ZPR9 expression increases MPK38 kinase activity in CRISPR/Cas9 ZPR9 knockout cells. Immunoprecipitated MPK38 (IP:α-MPK38) was assayed for its kinase activity using recombinant kinase-dead (KD) ASK1, Smad3, or p53 protein as the substrate in the presence or absence of a MPK38-specific inhibitor OTSSP167 (1 μM, 2 h). For rescue experiments, ZPR9 KO HEK293 cells were transfected with ~3 μg of ZPR9 (KO + ZPR9). WT, wild-type; ^{32}P, ^{32}P incorporation; IP, immunoprecipitation; WB, Western blot; re., recombinant; KO, knockout.

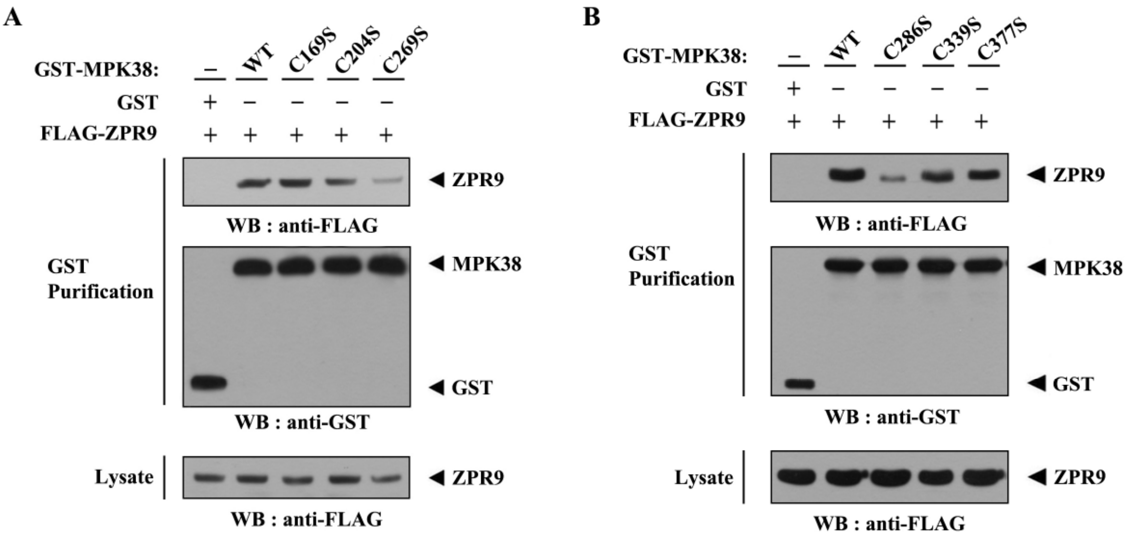

Supplementary Figure S2. Identification of potential cysteine residues of MPK38 for ZPR9 binding. (**A** and **B**) HEK293 cells, transfected with the GST-tagged expression vectors indicated, were lysed and the GST precipitates (GST purification) were analyzed for MPK38-ZPR9 complex formation by immunoblot analysis using an anti-FLAG antibody.

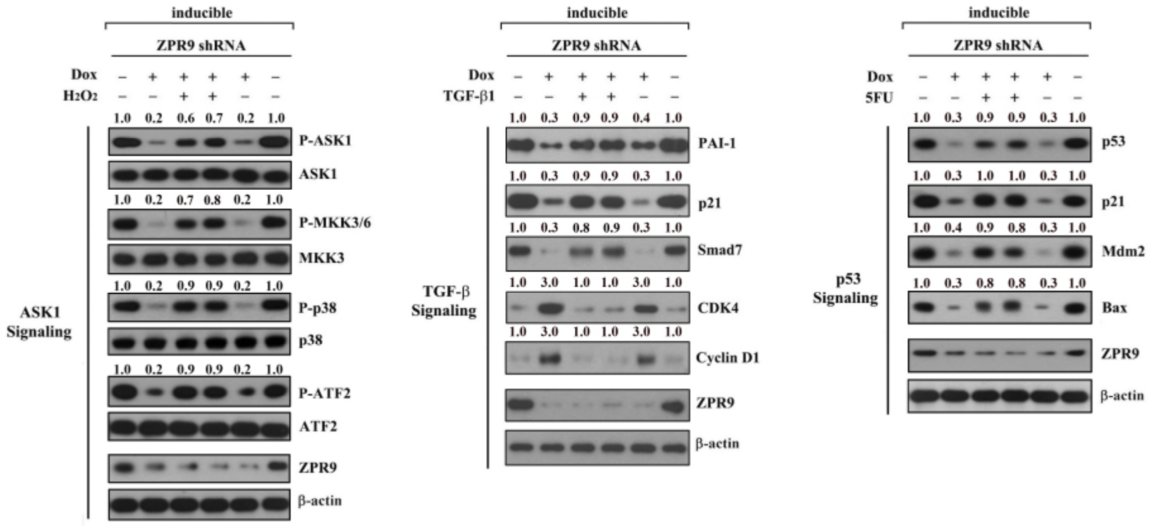

Supplementary Figure S3. Downregulation of ASK1/TGF-β/p53 signaling activation upon ZPR9 knockdown. NIH 3T3 cells harboring stably integrated pSingle-tTS-shRNA containing a ZPR9-specific shRNA (inducible ZPR9 shRNA) were treated with (+) or without (-) H_2O_2 (2 mM, 30 min), TGF-β1 (2 ng/ml, 20 h), or 5FU (0.38 mM, 30 h) in the presence or absence of doxycycline (Dox: 1 μg/ml, 72 h) and then analyzed by immunoblot analysis using the indicated antibodies or the anti-phospho-specific antibodies shown in Figs. 5-7.

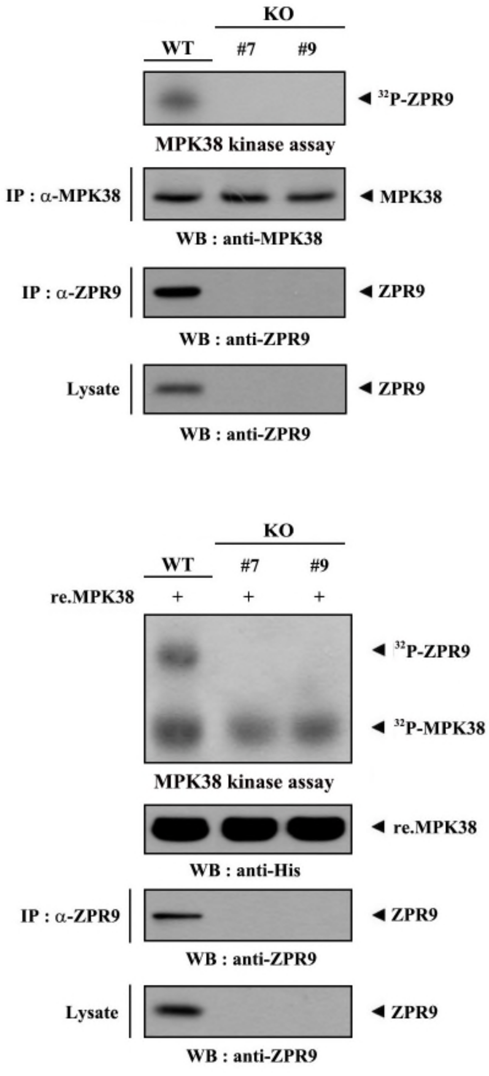

Supplementary Figure S4. Analysis of MPK38 kinase activity in CRISPR/Cas9 ZPR9 knockout cells. Immunoprecipitated MPK38 (upper panels) or recombinant MPK38 (lower panels) was assayed for its kinase activity in the presence of ZPR9 immunoprecipitates obtained from cell lysates of WT and clonal CRISPR/Cas9 ZPR9 KO isolates (clones #7 and #9) as the substrate.

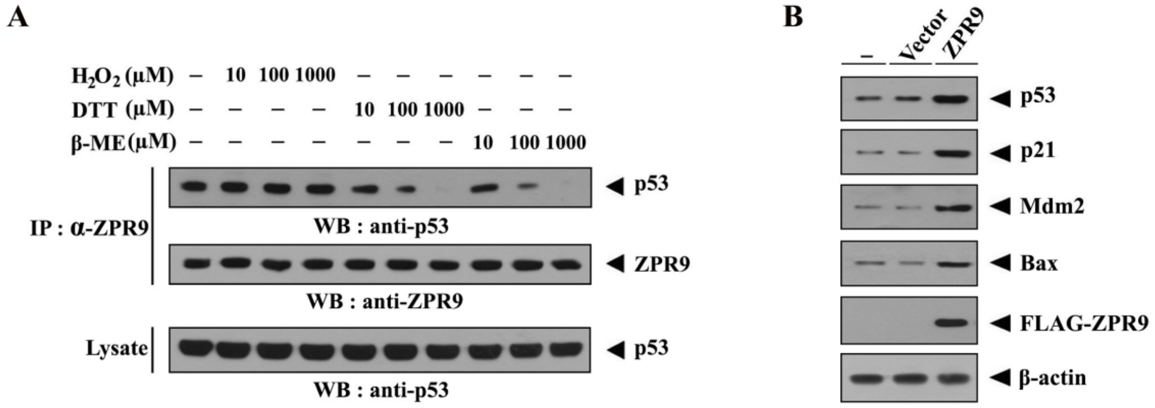

Supplementary Figure S5. Stimulation of p53 signaling by ZPR9. (**A**) Redox-dependency of the endogenous ZPR9-p53 interaction. HEK293 cell lysates were treated with the indicated concentrations of H_2O_2, DTT, and β-ME on ice for 0.5-1 h and then subjected to immunoprecipitation using an anti-ZPR9 antibody (IP:α-ZPR9). Immune complexes were analyzed for the presence of p53 by immunoblot analysis using an anti-p53 antibody. (**B**) Regulation of endogenous p53 targets by ZPR9. MCF7 cells were transfected with FLAG-tagged ZPR9, as well as an empty vector as a control, and the expression of p53 targets (p53, p21, Mdm2, and Bax) was determined by immunoblot analysis with antibodies specific to each protein. β-actin was used as a loading control.

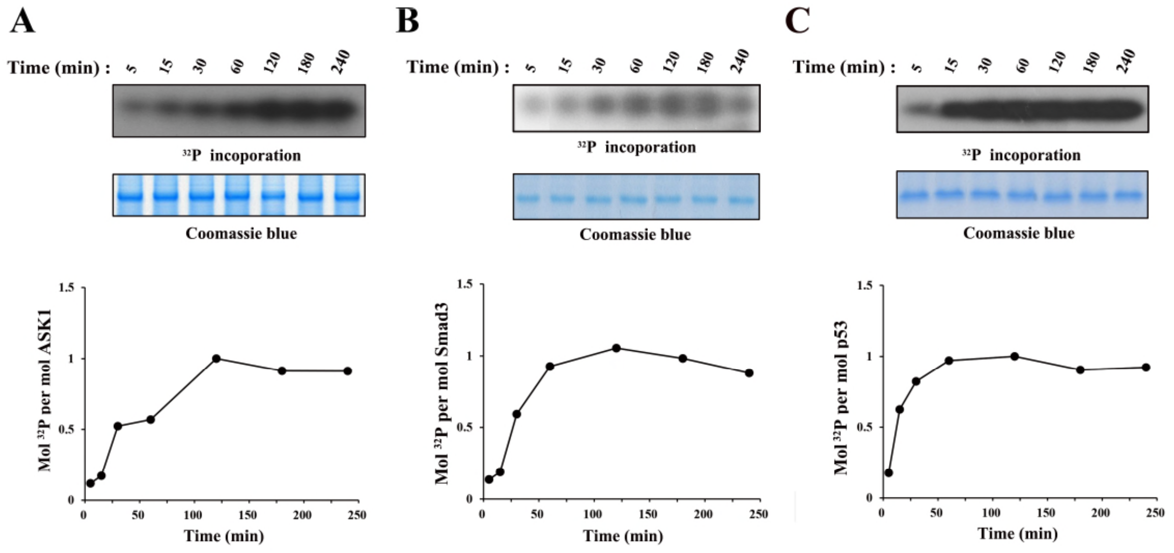

Supplementary Figure S6. Stoichiometry of MPK38 phosphorylation of ASK1, Smad3, and p53. Three different substrates (ASK1, Smad3, and p53) were phosphorylated by MPK38 for the indicated time periods, respectively. The *in vitro* kinase reactions were terminated by the addition of SDS sample buffer, the products were separated by SDS-PAGE and exposed to X-ray film for ~30 min. The equal loading of respective substrates was determined by Coomassie staining. Gel slices containing labelled ASK1 (**A**), Smad3 (**B**), and p53 (**C**) were excised and the amount of radioactivity was measured by the liquid scintillation counting, and the moles of ^{32}P incorporated per mole respective substrates were calculated and plotted as a function of time as described[33].
